# Home visitation program effectiveness and the influence of community behavioral norms: a propensity score matched analysis of prenatal smoking cessation

**DOI:** 10.1186/1471-2458-12-1016

**Published:** 2012-11-21

**Authors:** Meredith Matone, Amanda LR O'Reilly, Xianqun Luan, Russell Localio, David M Rubin

**Affiliations:** 1 PolicyLab, The Children's Hospital of Philadelphia, Philadelphia, PA, USA; 2 Division of General Pediatrics, The Children's Hospital of Philadelphia, 3535 Market, Room 1536, 34th Street and Civic Center Blvd, Philadelphia, PA 19104, USA; 3 Department of Pediatrics, University of Pennsylvania School of Medicine, Philadelphia, PA, USA; 4 Department of Biostatistics, University of Pennsylvania School of Medicine, Philadelphia, PA, USA

**Keywords:** Home visitation, Community context, Implementation, Evaluation, Smoking cessation

## Abstract

**Background:**

The influence of community context on the effectiveness of evidence-based maternal and child home visitation programs following implementation is poorly understood. This study compared prenatal smoking cessation between home visitation program recipients and local-area comparison women across 24 implementation sites within one state, while also estimating the independent effect of community smoking norms on smoking cessation behavior.

**Methods:**

Retrospective cohort design using propensity score matching of Nurse-Family Partnership (NFP) clients and local-area matched comparison women who smoked cigarettes in the first trimester of pregnancy. Birth certificate data were used to classify smoking status. The main outcome measure was smoking cessation in the third trimester of pregnancy. Multivariable logistic regression analysis examined, over two time periods, the association of NFP exposure and the association of baseline county prenatal smoking rate on prenatal smoking cessation.

**Results:**

The association of NFP participation and prenatal smoking cessation was stronger in a later implementation period (35.5% for NFP clients vs. 27.5% for comparison women, p < 0.001) than in an earlier implementation period (28.4% vs. 25.8%, p = 0.114). Cessation was also negatively associated with county prenatal smoking rate, controlling for NFP program effect, (OR = 0.84 per 5 percentage point change in county smoking rate, p = 0.002).

**Conclusions:**

Following a statewide implementation, program recipients of NFP demonstrated increased smoking cessation compared to comparison women, with a stronger program effect in later years. The significant association of county smoking rate with cessation suggests that community behavioral norms may present a challenge for evidence-based programs as models are translated into diverse communities.

## Background

Evidence-based maternal and child home visitation programs are currently undergoing widespread dissemination to a diversity of communities in nearly every state across the United States. The expeditious dissemination follows federal investment in the 2010 Affordable Care Act Maternal, Infant and Early Childhood Home Visitation Program, which boosted a decade-long trend of expansion of home visitation programs. However, owing the recency of significant investments in such programs, coupled with the complexity of home visitation interventions, there are insufficient data for continued effectiveness of these programs following replication. As such, there is a need for robust program evaluation efforts to understand the impact of replication on evidence-based programs and identify quality improvement needs.

One of the most widely disseminated home visitation programs is the Nurse-Family Partnership (NFP), a program of prenatal and postpartum home visitation by nurses for low-income, first-time families. NFP currently serves families in 42 states within the United States [1]. The NFP model has been evaluated in three randomized trials that have demonstrated positive program effects across a range of maternal outcomes, including prenatal smoking cessation, lengthened interbirth intervals, and reduced welfare receipt [2-4]. Child outcomes include a reduction in childhood injury rates, [5-9] improvements in school readiness, and a reduction in antisocial behaviors among adolescents born to program recipients [10,11].

In the state of Pennsylvania, NFP is widely disseminated, with 24 sites operating across the state. Recent evaluation of NFP in Pennsylvania found significant variation in program effects across replication sites on two program outcomes, pregnancy spacing and childhood injury [12,13]. The results of these analyses, which indicate that large heterogeneity in program effect exists among sites, lay the groundwork for further examination of the determinants of program success at a site-level. This question is a central topic in the fields of translational and implementation research which espouse both model fidelity and flexibility to community context in the successful dissemination of evidence-based interventions [14-19].

The influence of context, particularly, behavioral norms, on smoking cessation behaviors has been well documented. Such research has demonstrated higher cessation behaviors among adults residing in communities with low smoking prevalence and among adolescents with a low proportion of peers with smoking behaviors [20,21]. Additionally, perceived strong antismoking community norms have been associated with cessation [22]. However, research examining the relationship of community behavioral norms to smoking cessation program success is limited. Given that prenatal smoking rates in Pennsylvania vary greatly, from 7% to greater than 40% across PA counties, [23], the state provided an opportunity to test the hypothesis that program recipients who reside in communities with high smoking prevalence versus those residing in communities with lower prevalence might be less likely to quit smoking despite receipt of the same cessation intervention. Detecting such an effect, independent of the program effect, would help to characterize the challenge raised by community context on the success of implementation.

In view of large diversity in smoking rates across communities served by NFP in the state, this study sought to investigate whether cessation was associated with community smoking patterns, with the aim of contributing to the translational and implementation literature as it relates to home visitation. As such, the study objectives were twofold: 1) to build on prior work evaluating a home visitation program following large-scale replication by examining prenatal smoking cessation outcomes, and 2) to test the hypothesis that community norms negatively affect program outcomes.

## Methods

The primary sources of data covering a period of January 1, 2003 to December 31, 2007 were: (1) the enrollment history of clients participating in 24 NFP programs throughout Pennsylvania; (2) birth certificate files from the Pennsylvania Department of Public Health; (3) death certificate files from the Pennsylvania Department of Public Health; and (4) welfare eligibility files from the Department of Public Welfare.

The target population was clients from the 24 NFP sites in Pennsylvania who were enrolled in the NFP program between January 1, 2003 and December 31, 2007. Included were women who: (1) delivered a first-born infant; (2) self-reported tobacco cigarette use in the first trimester of pregnancy on birth certificate; and (3) received welfare assistance from the Commonwealth of Pennsylvania within 12 months prior to the birth of their first-born infant.

Eligible women for an unexposed comparison group were identified following a previously described linkage to birth certificate and welfare eligibility data from women residing in NFP communities who met the inclusion criteria noted above [13]. The unexposed comparison group identified women eligible for enrollment in NFP who were non-enrollees. Among comparison women, reasons for non-enrollment in NFP were not determinable at an individual-level, but likely include: program caps on yearly enrollment, receipt of alternate community services, and lack of interest or ability to enroll. To identify a comparison group from among the unexposed eligible women, a propensity score analysis used data from birth certificates and welfare eligibility files to model factors associated with a woman’s participation in NFP. The factors included maternal education (<12th, high school, some college or higher), maternal race (White, Black, Hispanic, Other), marital status (y/n), TANF receipt prior and/or during first trimester of pregnancy (y/n), foodstamp receipt prior and/or during first trimester of pregnancy (y/n), and history of gestational diabetes or hypertension (y/n). In addition, variables were included that encoded high density zipcodes within each site catchment area in order to drive the selection of unexposed comparison women toward high-penetration neighborhoods of interest. This density variable was created by identifying zipcodes as high density that enrolled more than 5% of the NFP client population within the site catchment area. Finally, models were stratified on maternal age (≤18 years, >18 years) and time period of birth cohort (2003–2005, 2006–2007) to force balancing on these factors for subsequent stratified analyses.

We developed a separate propensity score for each site to allow for the maximum flexibility in modeling of potential confounders with the fewest assumptions about the association of covariates and NFP participation across sites. Using a separate logistic regression for each site, the expected probability of participation in NFP (propensity score) was then estimated based on the above characteristics for each woman within a site [24,25]. The next step excluded as potential matches all unexposed comparison women who had propensity scores that fell outside the range of propensity scores of NFP clients. This initial exclusion left a group of unexposed comparison women and a group of NFP clients who shared propensity scores with “common support” – or overlapping ranges of propensity scores. Using a caliper of 0.05 (probability scale), one or more unexposed comparison women were selected using a nearest neighbor match without resampling (up to a maximum of 4 matched comparison women per client). Matching was done with the program gmatch under the SAS® Statistical Package v9.1.3 [26]. To avoid bias in differential matching rates across clients, analysis weights were assigned to comparison women based on the number of unexposed women matched to the NFP client [27]. Overall, successful balance of covariates was achieved within each site propensity score model. There were several exceptions, principally stemming from small sample size in which any single woman was able to shift weighted balance significantly. At a site model level, a threshold of ≥5% weighted case-comparison difference was used to identify potential covariate imbalance. Two agencies showed imbalance in the case-comparison weighted difference for race (10% and 13%), eight for education (range: 5-10%), five for prenatal foodstamp receipt (range: 5-9%), six for prenatal TANF receipt (range: 5-14%), and six for prenatal hypertension (range: 5-10%). In aggregate among all agencies, the study cohort was balanced across all covariates (see Table 1).

**Table 1:** **Characteristics of nurse-family partnership clients compared with all potential comparison women, welfare-eligible comparison women, and final matched comparison women** a**cross the commonwealth of Pennsylvania**

**Characteristics**	NFP Clients^*^	**NFP Smoking Clients**	**NFP Matched Clients**	All PA Births^**^	PA Welfare Eligible Births^**^	PA Welfare Eligible Smoking Comparisons^**^	Matched Comparisons^**^
Race, black	25%	12%	10%	19%	28%	13%	11%
<12th grade	50%	50%	49%	21%	34%	34%	45%
Smoking History+	38%	100%	100%	23%	34%	100%	100%
Young ≤18	44%	37%	34%	7%	25%	19%	30%
Urban	80%	74%	77%	89%	88%	85%	79%
Unmarried	90%	92%	91%	43%	83%	90%	91%
Foodstamp Receipt (first trimester)	44%	47%	45%	-	33%	34%	39%
TANF Receipt (first trimester)	41%	43%	41%	-	34%	35%	41%

^*^2003-2007.

^**^Births within NFP Service Regions (2003–2007).

+ Self-reported smoking prior to pregnancy and/or first trimester smoking.

- Data unavailable.

The primary outcome was a binary measure of smoking cessation in the third trimester of pregnancy. Smoking cessation was defined as self-reported use of zero cigarettes during the last three months of pregnancy as recorded on birth certificates. The two independent variables of interest were the assignment to the NFP program (yes/no) and a measure of the baseline county smoking rate where the site resided. Baseline county smoking rate was created for each client and was defined as the proportion of all women delivering in the county with birth certificate self-reported use of cigarettes in the first three months of pregnancy or in the three months prior to pregnancy where first trimester smoking information was missing (n = 308, 0.9%).

Multivariable logistic regression of the matched sample examined the association between smoking cessation and NFP participation. We hypothesized that smoking cessation success might also depend upon the prevalence of prenatal smoking in a woman’s community. Because prenatal smoking rates at the county level varied within site catchment area, we decomposed this factor into the site-level mean (to measure the across-site component) and the difference between mother’s zipcode prenatal smoking rate (calculated by estimating prevalence within zipcode) and the county rate (to measure the within site component) [28]. We estimated and report robust variance estimates to account for the potential of lack of model fit owing to possible overdispersion of the data. Results were expressed as odds ratios and predictive margins [29].

Analyses were conducted using Stata versions 11.0 (College Station, TX) and SAS v9.2 (SAS Institute, Cary NC). Approval for the study was granted by the Department of Public Welfare for the Commonwealth of Pennsylvania. Ethical approval was granted by the Institutional Review Board at the Children’s Hospital of Philadelphia.

## Results

From 2003–2007, 24 agencies enrolled 7276 clients. Eighty-one percent (5,909) of clients were welfare-eligible with a first singleton infant identified from Pennsylvania birth certificates. Following exclusion of 62% of clients with a non-smoking history (4,511), local-area propensity score matched 1,552 clients to 4,877 comparison women for a final study sample of 6,429 women.

The majority of the study cohort were unmarried (91%), resided in urban areas, and were not of Black race; one-third of the cohort was <18 years of age and nearly half had less than high school educational attainment. In comparison to all NFP clients, clients in this smoking cessation cohort were similar in educational achievement, urban/rural residence, marital status, and welfare reciept; however, the study cohort was more likely to be white and of older age (Table 1). After propensity score matching, selection factors were balanced between clients and comparison women.

NFP clients experienced significantly increased rates of smoking cessation when compared to matched comparison women (OR: 1.26, 95% CI: [1.11, 1.43], p = 0.002). While smoking cessation increased over time for both NFP clients and comparison women, the association of NFP participation and prenatal smoking cessation was stronger in a later implementation period (adjusted cessation rate: 35.5% for NFP clients vs. 27.5% for comparison women, p < 0.001, Table 2) than in an earlier implementation period (28.4% vs. 25.8%, p = 0.114).

**Table 2:** **Standardized probability of prenatal** smoking cessation by time period*

	***NFP Clients *% (95% CI)**	***Comparison Group *% (95% CI)**	***p value***
2003-2005	28.4 (24.8, 32.0)	25.8 (23.2, 28.4)	0.114
2006-2007	35.4 (31.1, 39.8)	27.5 (25.4, 29.6)	0.001

*Predictive margins standardized for county smoking rate.

Adjusting for NFP program effect, county smoking rate was significantly associated with prenatal smoking cessation, such that stronger community smoking prevalences were associated with reduced cessation behavior. Per 5-percentage point increase in county smoking rate, the odds of smoking cessation were reduced by 16% (OR: 0.84, 95% CI: [0.75, 0.95], p < 0.001).

## Discussion

This analysis found that following large-scale dissemination, the NFP program in Pennsylvania was able to achieve positive outcomes on prenatal smoking cessation. Smoking cessation outcomes improved over time, a finding that is consistent with prior home visitation research showing that time to effectiveness following implementation of evidence-based programs may be delayed [13]. These results suggest that prenatal smoking cessation continues to be a strong outcome for the NFP program.

At the same time, the effect of county smoking rate on the prenatal smoking cessation outcome underscores community behavioral norms as a potential contextual challenge of multi-site program dissemination efforts. It also affirms the importance of a context-focused research agenda, such as that offered by Greenhalgh et al. (2004) [30] for diffusion of health service innovations. In an era marked by increased dissemination of evidence-based public health programs, program evaluation efforts must prioritize context-informed process evaluations in order to strengthen the evidence base for the successful translation of such programs. Improving program translation will require a stronger appreciation of the levers by which community norms operate in complex, multi-faceted programs—specifically, implementation, fidelity, and client behavior. In this analysis, for example, this would mean disaggregating the effect of county smoking rate on smoking cessation into its component parts, which might include: the relationship of community norms and smoking behaviors at the client-level; the relationship of community norms and smoking behaviors at the nurse home visitor-level; and the relationship of community norms and program adaptation at the site-level.

At a program level, routine process evaluation efforts that consider contextual information might offer operational benefit. This information can serve as an important frame of reference for the interpretation of outcome data and consequently, a critical resource for targeted quality improvement efforts. Additionally, contextual information can inform program benchmarks. In this analysis, because the baseline differences in community smoking prevalence was also associated with cessation, standardized benchmarks for smoking cessation across NFP sites might overidentify need in well-performing sites located in high smoking communities and underidentify need in poor-performing sites located in lower smoking communities. Site-specific benchmarks for evidence-based programs may be an efficient quality improvement tool.

This study has several limitations. The first is the use of self-reported prenatal smoking birth certificate data. Self-reported smoking on birth certificates has been found to underrepresent actual smoking behaviors. However, the estimates of smoking prevalence in this study sample may be less vulnerable to underrepresentation, as research has demonstrated that Medicaid-enrolled women, younger women, and women with less educational attainment are less likely to misreport smoking behaviors on birth certificates [31,32]. Additionally, this analysis was intentionally restricted to years following the 2003 birth certificate revision to include trimester-specific smoking behaviors, as this revision has been found to have less misreport [33]. Missing data limited the use of smoking intensity information, as self-reported number of cigarettes, into study analyses. As intensity of smoking behavior is likely associated with cessation behaviors, future studies should aim to assess the relationship between intensity and cessation.

The observational study design also represents a limitation, as the introduction of selection bias is a prominent concern in observational studies. The use of propensity score matching to control for measured differences between NFP mothers and the comparison group minimizes this concern; however, unmeasured differences may still exist given the limitations of administrative data to measure maternal characteristics, resulting in residual confounding. In spite of selection bias limitations, observational study designs utilizing propensity score matching represent a valuable method for large-scale program evaluation following dissemination, as the utility and practicality of experimental evaluation designs in public health program dissemination are limited [34-36].

## Conclusions

This evaluation of prenatal smoking cessation within an evidence-based home visitation model demonstrates continued effectiveness following large-scale dissemination. The results of this analysis also demonstrate a relationship between context and program outcomes. The implication is that in certain settings, community norms might compromise program success in spite of proper implementation and fidelity efforts. As such, process evaluation will be an important tool for buffering outcomes during program translation into diverse communities.

## Competing interests

The authors declare that they have no competing interests.

## Authors’ contributions

MM, DR, and RL contributed to the study concept and design, statistical analysis and interpretation of data, and drafting and revision of the manuscript. AO and XL contributed to study concept and design and acquisition of data. All authors read and approved the final manuscript.

## Role of the sponsor

The Pennsylvania Department of Public Welfare had no role in the design and conduct of the study; collection, management, analysis, and interpretation of the data; and preparation, review, or approval of the manuscript.

## Pre-publication history

The pre-publication history for this paper can be accessed here:

http://www.biomedcentral.com/1471-2458/12/1016/prepub
